# Experimental and clinical studies on pharmacological actions of the genus *Achillea*: A comprehensive and updated review

**DOI:** 10.22038/AJP.2024.23711

**Published:** 2024

**Authors:** Saeideh Saadat, Mojgan Rajabi, Mohammad Hossein Boskabady

**Affiliations:** 1 *Department of Physiology, School of Medicine, Zahedan University of Medical Sciences, Zahedan, Iran*; 2 *Applied Biomedical Research Center, Mashhad University of Medical Sciences, Mashhad, Iran*; 3 *Department of Physiology, Faculty of Medicine, Tabriz University of Medical Sciences, Tabriz, Iran*; 4 *Department of Physiology, Faculty of Medicine, Mashhad University of Medical Sciences, Mashhad, Iran*

**Keywords:** Achillea, Bioactive compounds, Pharmacological effects, Molecular mechanisms

## Abstract

**Objective:**

Species of the genus *Achillea* (from the family Compositae or Asteraceae) are widely used for their numerous pharmacological properties. The present paper reviews pharmacological actions and their possible underlying molecular mechanisms reported for various species of *Achillea*.

**Materials and Methods:**

Various databases including PubMed, Science Direct, and Scopus were used.

**Results:**

Immunosuppressive, anti-inflammatory and anti-oxidant effects were shown for these plants. In addition, it was shown that these plants pose wound-healing properties and antimicrobial effects on various bacteria as well as antitumor effects on different cell lines. *Achillea* species showed anti-arrhythmic, anti-thrombotic, vasorelaxant, anti-hyperlipidemic, anti-hypertensive, hepatoprotective and gastroprotective effects. In addition, the plants showed different endocrine effects such as anti-diabetic, estrogenic and anti-spermatogenic properties. Neurological effects of the plants also included anti-nociceptive and anti-anxiety actions. Clinical studies also indicated therapeutic effect of *A. millefolium* on multiple sclerosis, chemotherapy-induced oral mucositis in cancer patients, and dysmenorrhea but did not affect atopic dermatitis.

**Conclusion:**

*Achillea* species could be of therapeutic potential for treating of a wide range of diseases but further investigations are needed regarding the other properties of *Achillea* plants.

## Introduction

The genus *Achillea* from the family Asteraceae (also known as Compositae) (Saeidnia et al., 2011), includes 110–140 species (Applequist and Moerman, 2011) distributed mostly in Europe and Asia (Al-Snafi, 2013). The genus *Achillea* in Iran has seven endemic species out of nineteen identified species (Sharafzadeh et al., 2013) with popular name of “*Bumadaran*” (Saeidnia et al., 2011).

*Achillea* genus species have many pharmacological properties such as antimicrobial, anti-inflammatory, anti-allergic, anti-oxidant (Sharafzadeh et al., 2013), antispasmodic, anti-diabetic, anti-ulcer, antitumor, choleretic and hepatoprotective activities (Al-Snafi, 2013). The plants have also been used as diaphoretic, diuretic (Saeidnia et al., 2011), and cytotoxic agents (Al-Snafi, 2013). *Achillea* species have been employed in Persian traditional medicine for pneumonia, rheumatic pain, hemorrhage, and wound healing (Saeidnia et al., 2011).

There are few review papers on *Achillea* as a unique medicinal genus. Previously, the studies provided a brief overview on traditional and folk medical usage, phytochemistry, and biological activities including anti-human pathogenic, anti-phytopathogenic and anti-fish-pathogenic properties, as well as antioxidant, anti-cancer (Salehi et al., 2020), wound-healing, esterogenic, anti-diabetic, antispermatogenic, antiulcer, cytotoxicity, immunosuppressive, biological, antispasmodic and anti-inflammatory activities (Saeidnia et al., 2011) of *Achillea* plants without considering the molecular mechanisms. The present study reviews various pharmacological actions of various species of *Achillea* and their possible underlying molecular mechanisms, based on the scientific *in vitro*, *in vivo* or clinical studies.

## Materials and Methods

This review article is a summary of the existing literature regarding the pharmacological effects of the genus *Achillea*. The scientific databases namely, PubMed, Science Direct, Scopus, Medline and Google Scholar were searched for original articles published from 1969 to 2022 using the keywords such as genus *Achillea*, medicinal plants and their constituents to identify studies done to elicit the pharmacological properties of the genus *Achillea*.

## Results

### Chemical constituents

The pharmacological properties of *Achillea* may be attributed to its various secondary active metabolites. These metabolites include phenolic acids, flavonoids, terpenoids, coumarins, and sterols. Many studies have reported the chemical composition of *Achillea* species.

Terpenoids can be classified into monoterpenes, sesquiterpenes, diterpenes, and triterpenes. Some examples of diterpenes are three kaurane oxides isolated from *A. clypeolat*. *A. odorata* contain two triterpenes, Achilleol A and achilleol B, the main components of *Achillea* essential oils are monoterpenes (Si et al., 2006). Sesquiterpenes include guaianolides (12,6a-lactones and some 3-oxa (furan) derivatives), eudesmanes, germacranes and bisabolanes (isolated only from *A. cretica*). *Achillea* also contain an elemane, an oplopane, a cyperane, two longipinanes, two aliphatic sesquiterpenes and a 5, 6-seco-caryophyllan (Si et al., 2006).

In 1961, the first flavonoids, cynaroside and cosmosiin, were isolated from *A.* *millefolium* which showed spasmolytic activity (Si et al., 2006). In 1972, the germacrane ageratriol was isolated from *A.* *ageratum* L. (Si et al., 2006). In 1978, the structure of achillicin, the first natural proazulene found in the genus *Achillea* isolated and elucidated (Banh-Nhu et al., 1979). More recently, in 2006, Si et al. published a review article which presented the structures of the known phytochemical constituents of *Achillea* along with a brief description of their biological properties (Si et al., 2006). From a phytochemical perspective, various compounds including terpenoids, lignans, flavonoids, amino acid derivatives, and a small number of other compounds uch as fatty acids, alkanes, and inulin, were extracted and characterized in *Achillea* species (Si et al., 2006).

**Figure 1:**
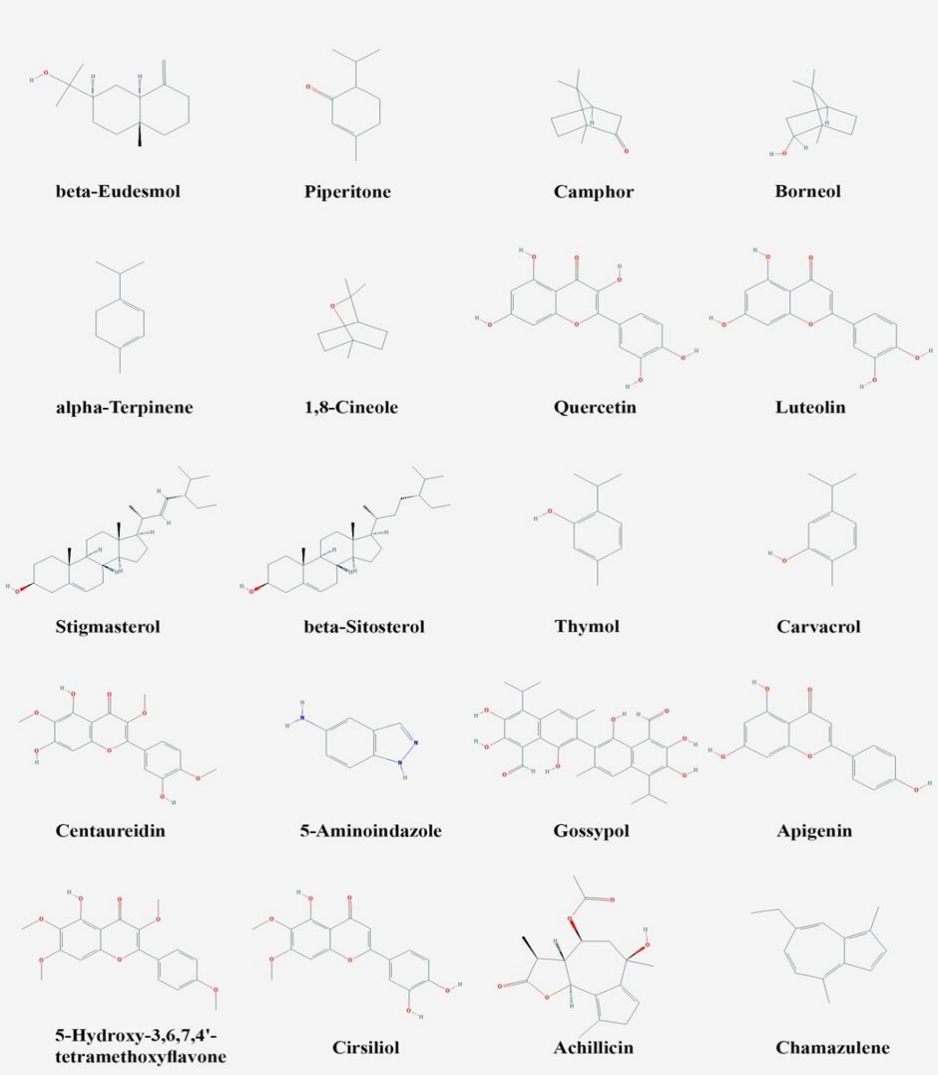
Chemical structures of some of the important compounds of *Achillea* species known for pharmacological actions. Sesquiterpenoid beta-eudesmol, piperitone, camphor, borneol and alpha-terpinene isolated from *A. biebersteinii*; 1,8-cineole isolated from *A. biebersteinii*, *A. setacea* and* A. wilhelmsii*; quercetin isolated from *A. talagonica*; luteolin isolated from *A. talagonica* and *A. millefolium*; stigmasterol and beta-sitosterol isolated from *A. ageratum *and* A. ageratum*; thymol and carvacrol isolated from *A. wilhelmsii; *centaureidin isolated from *A. clavennae *and* A. millefolium; *5-aminoindazole, gossypol, apigenin, achillicin and chamazulene isolated from *A. millefolium; *5-hydroxy-3,6,7,4′-tetramethoxyflavone isolated from *A. *nobilis; and Cirsiliol isolated from *A. fragrantissima*.

The essential oil of *A. santolina* comprises a total of 54 constituents. Predominant among these constituents are 1, 8-cineole, fragranol, fragranyl acetate, and terpinen-4-ol (Al-Snafi, 2013). *A. kellalensis* contains various monoterpenoids such as camphor (34.0%), borneol (12.6%), α-thujone, cineol, bornyl acetate and camphene (Rustaiyan et al., 1999) and root of *A. clypeolata* contains various diterpenoids such as 16α,17-epoxy-ent-kaurane, 3α-acetoxy-16α,17-epoxy-ent-kaurane and 19-acetoxy-16α,17-epoxy-ent-kaurane (Aljančić et al., 1996). The investigation of the chemical composition of *A. wilhelmsii* demonstrated the existence of 30 compounds, accounting for 94.48% of the total oil, with a yield of 0.82% w/w. The primary constituents of the oil were α-thujene, α-pinene, sabinene, p-cymene, 1,8-cineole, linalool, camphor, thymol, and carvacrol (Boskabady et al., 2009; Kazemi and Rostami, 2015). The main constituents of these plants and pharmacologically important chemical compounds present in *Achillea* species are shown in Table 1 and Figure 1, respectively.

### Antitumor, antimicrobial and wound-healing effects

#### Antitumor effects

Cytotoxic effects of *A. clavennae* and its constituents were shown in several studies such as cytotoxic effect of centaureidin in a tumor assay (Si et al., 2006). Guaianolides, 9α-acetoxyartecanin XVII and apressin XVIII isolated from the aerial part of *A. clavennae* showed cytotoxic effects against HeLa, K562 and Fem-X human cancer cell lines but a bisabolene, inducumenone XIX exhibited a moderate activity and a flavonol, centaureidin XX was the most active compound (Trifunović et al., 2006). Tanaphillin XIV, 3β-methoxy-iso-seco-tanapartholide XIII, iso-seco-tanapartholide XV, and 8-hydroxy-3-methoxy-iso-seco-tanaparatholide XVI isolated from *A. falcata*, inhibited HaCaT-cell growth and reduced keratinocyte cell viability (Ghantous et al., 2009). The anti-proliferative effects of various extracts from *A. millefolium* on three human tumor cell lines (HeLa, MCF-7 and A431) showed that the chloroform extract has a strong inhibitory activity on HeLa and MCF-7 cells and casticin and paulitin were highly effective against all three tumor cell lines (Csupor‐Löffler et al., 2009b).

More prominent growth inhibition of the chloroform extract from *A. ageratum* and its derivatives, stigmasterol and β-sitosterol against Hep-2 and McCoy cells compared to 6-mercaptopurine against both cell lines was observed (Gómez et al., 2001).

The ethanol extract of *A. millefolium* was more cytotoxic on MCF-7 breast cancer cells and the flower extract showed a higher antiproliferative effect (Amini Navaie et al., 2015). The chloroform-soluble extract of *A. millefolium *showed high inhibitory activities on HeLa and MCF-7 cells (Csupor‐Löffler et al., 2009a). In human cervical cancer (HeLa) cells, *A. millefolium *ethyl acetate fraction induced apoptosis and cell cycle arrest (Abou Baker, 2020) and hydroalcoholic extract of *A. wilhelmsii* decreased cell death-associated gene expression while causing DNA damage (Sargazi et al., 2020).

Hydrodistillation extract of *A. fragrantissima* showed an IC50 value of 0.51 µg/ml for MCF-7 and 0.62 µg/ml for HCT116 but the oil prepared by volatile solvent indicated an IC50 value of 0.80 µg/ml for MCF-7 and 0.91 µg/ml for HCT116 and the cytotoxic activity of the essential oil may be due to the synergistic effect of its constituents (Choucry, 2017). In A2780 ovarian cancer cells, 1,8-cineole demonstrated cytotoxicity and it was more selective against MRC5 cells, promoted apoptosis in A2780 cells and increased preG1 events (Abdalla et al., 2020).

#### Antimicrobial effects

*A. damascena* methanolic extract showed inhibitory effect against *Proteus* species, *Candida albicans*, *Staphylococcus aureus*, *Shigella dysenteriae*, *Salmonella enteritidis*, and *Streptococcus faecalis* (Barbour et al., 2004).

The extracts of the aerial parts of *A. clavennae*, *A. holosericea*, *A. lingulata* and *A. millefolium* exhibited antimicrobial activities against some fungi and bacteria and the extract of *A. clavennae* also showed potent antimicrobial activity (Stojanović et al., 2005).

The methanolic extract and essential oil of *A. millefolium* showed antimicrobial activity effect against several microorganisms casing lung infection but water-insoluble parts of the methanolic extracts exhibited slight or no antimicrobial activity (Akram, 2013). Antimicrobial effects of borneol have also been reported previously (Candan et al., 2003; Daniel et al., 2020).

Antimicrobial properties of* A. biebersteinii* and *A. santolina*, were also reported against *Staphylococcus aureus*, *Pseudomonas aeruginosa*, and *Candida albicans* and the extract of *A. biebersteinii* from Jordan was effective against *S. aureus* at 10 ppm (Khalil et al., 2009). However, the extract of *A. biebersteinii* from Turkey inhibited *S. aureus* at 300 ppm. In this study, essential oil of *A. biebersteinii* exhibited antimicrobial effect against 14 fungi and 8 bacteria whereas methanolic extract was inactive (Bariş et al., 2006). *A. biebersteinii* essential oil showed anti- bacterial activity by causing a rise in the permeability of the cell membrane (Al-Shuneigat et al., 2020).

The extracts of *A. bierbersteinii* and *A. santolina* (60 ppm) inhibited *P. aeruginosa*, and alcoholic and oil extract of *A. falcata* inhibited the growth of *S. aureus* and *P. aeruginosa* indicating inhibitory effects of *Achillea* plants from Jordan on both Gram-positive and Gram-negative bacteria (Khalil et al., 2009). However, there was no effect for methanolic extracts of *A. santolina* on *Candida albicans*, *Candida glabrata*, or *Candida krusei* (Darwish and Aburjai, 2011).

The oils of *A. setacea* and *A. teretifolia* containing eucalyptol (1, 8-cineole) as their major constituent, inhibited *Acinetobacter lwoffii,* *Candida albicans *and* Clostridium perfringens*. Therefore, the constituents of these oils, camphor and its derivatives, borneol, terpinen-4-ol and eucalyptol (1,8-cineol) could be considered main antimicrobial agents (Unlu et al., 2002). *A. wilhelmsii* oil was also highly effective against *Escherichia coli* and *Candida albicans* (Kazemi and Rostami, 2015). Flower head from *A. gypsicola* harvested in the evening at seed maturation stage and leaf harvested in the evening at post flowering stage showed marked antimicrobial effects (Açıkgöz, 2020). Essential oils of different segments of *A. filipendulina* exhibited varying Gram‐positive and Gram‐negative antibacterial effects (Aminkhani et al., 2020).

#### Wound-healing effects

Several studies have reported the effects of medicinal plants on wound-healing process, including coagulation, inflammation, collagenation, fibroplasia, epithelization, and wound contraction (Pirbalouti et al., 2010).

Topical administration of aqueous extract of *A. kellalensis* flowers, locally known as “*Golberrenjas*” or “*Bumadaran-e-Sabzekohî*” (Rustaiyan et al., 1999) exhibited wound healing activity in rats (Pirbalouti et al., 2010). The n-hexane extract of *A. biebersteinii* showed strong activity in wound healing models. The activity of the plant may be due to a synergistic interaction among these compounds (Akkol et al., 2011). The methanol extract of the leaves of *A. eriophora* stimulated human fibroblast proliferation at low concentrations (0.1-0.8 µg/ml) and induced migration of the cells at intermediate concentrations (1-30 µg/ml) (Varasteh-Kojourian et al., 2017).

Topical treatment with ethanol extract of *A. asiatica* enhanced epithelialization and accelerated wound healing in rats. *A. asiatica* reduced nitric oxide (NO) and prostaglandin E2 (PGE2) level and mRNA expression of interleukin (IL)-6, tumor necrosis factor-alpha (TNF-α), IL-1β, and cyclooxygenase-2 (COX-2). Furthermore, *A. asiatica* increased collagen expression in Hs68 fibroblasts through activating transforming growth factor-β (TGF-β), keratinocyte differentiation and motility via inducing keratinocyte, β-catenin, and Akt differentiation markers. Luteolin and apigenin were found to be responsible for these effects (Dorjsembe et al., 2017).

In a double-blind clinical trial study, *A. millefolium* ointment reduced perineal pain, redness, ecchymosis and edema of episiotomy wound (Hajhashemi et al., 2018). Daily topical application of *A. millefolium* extract in rabbits with full-thickness skin defects, accelerated wound healing (Temamogullari et al., 2009). The hydroalcoholic extract of *A. millefolium* showed considerable potential for wound healing in rabbits which was possibly mediated through acceleration of the collagenation and proliferation phase of wound healing (Hemmati et al., 2002). Aqueous and alcoholic extracts of *A. millefolium* leaves improved wound healing in rats, by increasing wound contraction rate, granulation tissue dry weight and wet weight content, and skin breaking strength (Nirmala and Karthiyayini, 2011). The wound-healing activity is most probably a result of the synergistic effect of the plant extract derivatives and additive effect of hiperisin. Anti-inflammatory, anti-oxidant, immunosuppressive, antitumor, antimicrobial and wound-healing effects of *Achillea* species plants are summarized in Table 2.

#### Smooth muscle relaxant effect

Total extract of *A. nobilis* subsp. Sipylea has shown antispasmodic activity in rat duodenum. It was suggested that this effect was probably elicited through the inhibition of calcium entry into the cell cytoplasm associated with disrupting the biochemical mechanisms (Karamenderes and Apaydin, 2003).

The spasmolytic effect of *A.* *millefolium* has been suggested to be due to the flavonoids constituents of the plant (Chandler et al., 1982). Several derivatives of total flavonoids extracted from the aerial parts of *A. nobilis* (Kastner et al., 1995) showed spasmolytic properties in various smooth muscles (Harborne and Williams, 2000) by inhibiting tonic and phasic contractions in rat ileum (Hammad and Abdalla, 1997). They also decreased the smooth muscle tone of the main pulmonary artery and trachea of the guinea pig, and the uterus and vas deferens of the rat (Abdalla et al., 1989; Rojas et al., 1996; Van Den Broucke and Lemli, 1983). *A. fragrantissima* flavone, cirsiliol also showed rat ileum relaxation and inhibited maximal contractions (Mustafa et al., 1992). Eupatilin XXXV, galangin XXXIV and quercetin XXI, the derivatives of *Achillea*, also relaxed the ileum (Hammad and Abdalla, 1997).

Hydroalcoholic extract of *A.* *millefolium* (1%) decreased the ileum smooth muscle contractions induced by acetylcholine (1 μg/ml) and potassium chloride (60 mM). The results also suggested that the relaxing effect of the extract is due to flavonoids constituents of the plant specially quercetin and apigenin (Sedighi et al., 2013). In rabbit jejunum, the extract of *A. millefolium* (0.3–10 mg/ml) caused a concentration-dependent relaxant effect on spontaneous and K^+^-induced contractions and similar to verapamil, and shifted Ca^2+^ concentration-response curves (CRCs) to the right, (Yaeesh et al., 2006).

*A. wilhelmsii* extract (2, 4, 6 and 8 mg/ml) showed significant relaxant effects on guinea pig tracheal smooth muscle (TSM) (Boskabady et al., 2009) by muscarinic inhibitory effect. The relaxant effect of cirsiliol on smooth muscle was also shown due to transmembrane Ca^2+^ influx inhibition (Mustafa et al., 1992). The relaxant effect of cirsiliol on smooth muscle was indicated to be induced by inhibiting calcium ions influx to the cell (Boskabady et al., 2009) and β-adrenergic and histamine (H_1_) receptors are not involved in the relaxant effect of *A.* *wilhelmsii* (Feizpour et al., 2013). *A. millefolium* aqueous-ethanol extract showed a competitive antagonistic effect at muscarinic receptors, a stimulatory effect on β2-adrenergic receptors, and a slight inhibitory effect on histamine (H1) receptors (Koushyar et al., 2013). *A. millefolium* hexanic extract showed relaxant effect on rat TSM, mainly due to calcium channel and NO release blockade (Arias-Durán et al., 2020). Carvacrol, a constituent of *A. wilhelmsii*, also showed relaxant effect on TSM which was not due to histamine H_1_, or muscarinic blocking or β_2_-adrenergic stimulatory effects (Boskabady and Jandaghi, 2003).

**Figure 2:**
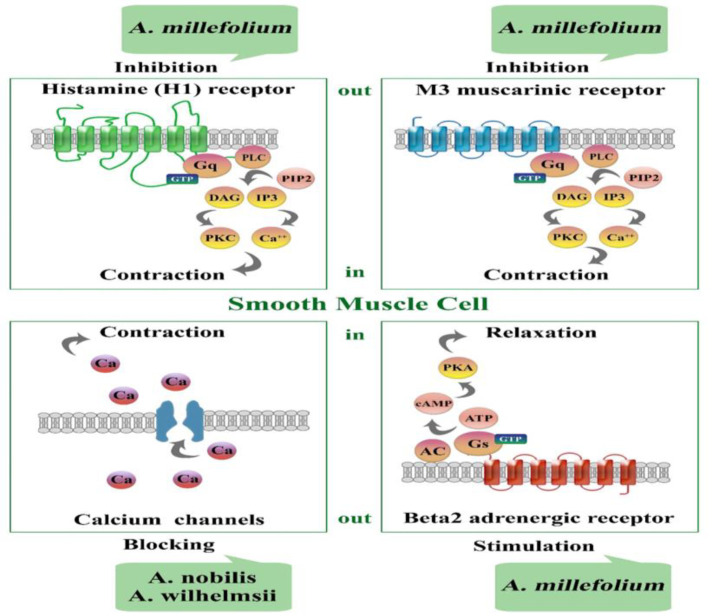
Possible molecular mechanisms of *Achillea* species on the tracheal smooth muscle. AC: adenylyl cyclase; ATP: adenosine triphosphate; cAMP: cyclic adenosine monophosphate; Ca^++^: calcium; DAG: diacylglycerol; GTP: guanosine triphosphate; IP3: inositol trisphosphate; PIP2: phosphatidylinositol biphosphate; PKA: protein kinase A; PKC: protein kinase C; and PLC: phospholipase C.

In different studies, the relaxation of guinea pig TSM and main pulmonary artery, and rat uterus and vas deferens was also observed for various plants (Abdalla et al., 1989; Rojas et al., 1996; Van Den Broucke and Lemli, 1983). Smooth muscle relaxant effects of *Achillea* species are shown in Table 3. In addition, the possible molecular mechanisms of *Achillea* species on TSM are shown in Figure 2.

### The effects on the digestive system

#### Treatment of alimentary-tract disease

There are some reports on gastrointestinal effects of *Achillea*, such as anti-ulcer, anti-bacterial, hepatoprotective, choleretic, and antispasmodic properties (Niazmand et al., 2010). In fact, *A.* *millefolium* has been used against digestive conditions and as a cholagogue agent. The utilization of herbal teas from various species of *A. millefolium* group, particularly for gastrointestinal diseases, is prevalent in traditional medicine (Si et al., 2006). *A. millefolium *and related species are commonly employed for treating diarrhea, abdominal pain, and stomachache in Turkish traditional medicine (Akram, 2013; Si et al., 2006).

#### Choleretic activity

Previous experiments showed that *A. millefolium* increased bile flow in a dose-dependent manner in isolated perfused rat liver. *A. millefolium* showed more pronounced choleretic effect than cynarin (1,3-DCCA), which is the main compound of *Cynara scolymus*. Simultaneous administration of dicaffeoylquinic acids (DCCAs) and luteolin, prepared from methanolic extract of *A. millefolium*, increased bile flow (Benedek et al., 2006).

#### Orexigenic effect

Treatment with hydro-alcoholic extract of *A. millefolium* (50 and 100 mg/kg, for 7 days) resulted in a positive dose-dependent effect on appetite in rats. However, it appears that the orexigenic effect of *A. millefolium* was not influenced by changes in ghrelin levels in the blood (Nematy et al., 2017).

#### Anti-ulcerogenic effects

Methanolic extract of *A. millefolium*, can cure *Helicobacter pylori*-induced stomach ulcer and gastritis (Mahady et al., 2005).

Low doses of* A. millefolium* (1 and 10 mg/kg) reduced acetic acid-induced chronic gastric ulcers, and increased gastric mucosa regeneration by increased cell proliferation, indicated by proliferating cell nuclear antigen (PCNA) immunohistochemistry. Treatment with *A. millefolium *improved glutathione (GSH) and superoxide dismutase (SOD) levels and inhibited the myeloperoxidase (MPO) activity in acetic acid-induced gastric lesions. Therefore, anti-oxidant properties *A.* *millefolium* may contribute to the gastroprotective activity of this plant (Potrich et al., 2010). The anti-ulcer potential of the aerial parts of the *A. millefolium* (0.3–1.2 g/kg/day, orally) with no signs of toxicity when administered for a long period, was shown (Cavalcanti et al., 2006).

Crude extract of *A. millefolium* leaves prevented ethanol and cold stress but not indomethacin-induced ulcers in rats. The antiulcer effect of *A. millefolium* is probably related to either inhibiting gastric secretion or increasing protective factors in the gastric mucosa (Baggio et al., 2002). The hydroalcoholic extract of *A. wilhelmsii* (1 and 2 mg/kg) in basal condition but not in vagotomized condition, increased acid output, indicating the inhibition of acid output by inhibiting the gastric vagal parasympathetic (Niazmand et al., 2010).

#### Hepatoprotective effects

Pre-treatment of mice with crude extract of *A. millefolium* prevented D-galactosamine and lipopolysaccharide (LPS)-induced rise in plasma alanine aminotransferase (ALT) and aspartate aminotransferase (AST). The extract improved architecture, parenchymal congestion, cellular swelling and apoptosis, indicating that the hepatoprotective effect of *A. millefolium* against D-galactosamine and LPS-induced hepatitis, through possible calcium channel blocking activity (Yaeesh et al., 2006). Smooth muscle relaxant effects of *Achillea* species plants and their effects on digestive system are shown in Table 3.

### The effects on the cardiovascular system

There are reports for cardiovascular effects of *A.* *millefolium* and this plant is prescribed in hypertension (Akram, 2013).

Various concentrations of *A. santolina* methanol extract on the electrophysiological properties of the heart reduced wenckebach cycle length, atrio-ventricular conduction, and effective refractory period (Khoori et al., 1999). *A. Santolina* showed a possible role in treating supraventricular tachyarrhythmia and vasoprotective activity of *A.* *millefolium* extract was also reported (Dall’Acqua et al., 2011). A histopathological study established the protective effect of ethanolic extract of *A. millefolium* on cisplatin-induced acute vascular injuries in the heart, liver and renal tissues (Eslamifar and Sabbagh, 2020).

The *in vitro* anti-aggregant and *in vivo* anti-thrombotic effect of extracts and fractions of *A.* *santolina* showed dose-dependent inhibition of platelet aggregation induced by collagen and adenosine diphosphate (ADP) *in vitro* (Al-Awwadi, 2010). However, the inhibition of experimental thrombosis was lower compared to other product of plant origin (Tohti et al., 2006; Umar et al., 2003), or non-steroidal anti-inflammatory drugs (NSAIDs) such as aspirin (Umar et al., 2004).

### Anti-hypertensive effects

In a clinical trial on hyperlipidemia and hypertension, patients were orally treated with either placebo or *A. wilhelmsii* extract (15-20 drops) for up to 6 months. Triglycerides decreased after 2 months, total cholesterol and LDL-cholesterol after 4 months, HDL-cholesterol after 6 months and blood pressure after 2 and 6 months (Asgary et al., 2000). In a rabbit model, the extract of *A. wilhelmsii* (80 mg/kg) decreased blood pressure (Niazmand et al., 2011) possibly due to its cardiac depressant and/or vasorelaxant effect as well as negative cardiac inotropic and chronotropic effects (Niazmand and Saberi, 2010).

The antispasmodic and vasorelaxant effects of carvacrol (Boskabady and Jandaghi, 2003; Can Baser, 2008; Peixoto‐Neves et al., 2010), luteolin (Jiang et al., 2005; Qian et al., 2010), apigenin (Jin et al., 2009), and 1, 8 cineole (Lahlou et al., 2002; Nascimento et al., 2009) as various constituents of *A.* *wilhelmsii* were indicated. Inhibition of Ca^2+^ channels, release from the intracellular Ca^2+^ stores, and activation of K^+^ channels contribute to the vasorelaxant effect of luteolin (Peixoto‐Neves et al., 2010). The vasorelaxant effect of *A. wilhelmsii* is mediated through the inhibition of extracellular influx of calcium ions via voltage and receptor-operated calcium channels (Niazmand et al., 2014). Table 4 summarizes the effects of *Achillea* species plants on the cardiovascular, endocrine and nervous systems.

### The effects on the endocrine system

#### Anti-diabetic effects

Hypoglycemic effect of *A. santolina* aqueous extract in streptozotocin-induced diabetic rats was shown (Al-Snafi, 2013). *A. santolina* treatment decreased blood glucose, serum NO, protein carbonyls (PCO), pancreatic MDA, and advanced oxidation protein (AOPP) levels but increased GSH, CAT and SOD levels. Therefore, *A. santolina* showed hypoglycemic effect perhaps due to its antioxidative potential (Yazdanparast et al., 2007).

#### Estrogenic effects

The estrogenic effects of dihydrodehydrodiconiferyl alcohol 9-O-beta-D-glucopyranoside, apigenin and luteolin, the derivatives of *A.* *millefolium *were reported (Innocenti et al., 2007). Apigenin has a weaker effect than the endogenous hormone on estrogen receptors-dependent pathways by activation of both α and β receptors but luteolin has a minimal effect on β receptor and does not activate α receptor (Innocenti et al., 2007).

#### Anti-spermatogenic effects

*A.* *millefolium* ethanolic extract exfoliated germ-cell necrosis, immature germ cells, and somniferous tubule vacuolization in mice and caused higher number of metaphases in the germ epithelium that might be due to cytotoxic substances or substances stimulating cell proliferation (Montanari et al., 1998). *A. millefolium* inflorescence hydroalcoholic extract showed a positive effect on sperm count, motility, and viability, and maturation of the nucleus (Karimpour et al., 2020).

Hydroalcoholic extract of *A. santolina* altered the seminiferous tubules histology including exfoliation of immature germ cells, disorganized germ epithelium, germ cell necrosis and metaphases number in germinal epithelium in mice. The potential anti-spermatogenic effects were suggested for *A. santolia* exerted (Golalipour et al., 2004). Table 4 summarizes the effects of *Achillea* species plants on the cardiovascular, endocrine and nervous systems.

### The effects on the nervous system and behavior

#### Anti-nociceptive effects

The traditional use of* A. millefolium* in muscular pain (Akram, 2013) and anti-nociceptive peripheral effect of *A.* *millefolium* known as analgesic drugs were reported (Pires et al., 2009). Polar fraction of *A. millefolium* extract administered intraperitoneally in rats showed higher sedation, pre-anesthetic and anti-anxiety effects than semi-polar, non-polar and diazepam (Rezaie and Ahmadizadeh, 2013).

Traditionally, *A. millefolium* has been applied as sleeping aids, probably acting through central adenosine mechanism, for sleep-inducing and sleep-maintaining effects (Schiller et al., 2006). Anxiolytic plants may affect either glutamic acid decarboxylase (GAD) or gamma-aminobutyric acid transaminase (GABA) transaminase and ultimately influence brain GABA levels and neurotransmission (Awad et al., 2007). *A. millefolium* inhibited GAD activity (Awad et al., 2007) and induced ionotropic response (Aoshima et al., 2006). Pentobarbital-induced sleeping was prolonged by *A. millefolium*-derived α-acids (Zanoli et al., 2005).

In the marble-burying test and elevated plus-maze, anxiolytic-like effects of *A. millefolium* (acute and chronic administration) at doses that did not affect locomotor activity were shown to be similar to the effects of diazepam which was not influenced by picrotoxin, but was partially inhibited by flumazenil. Therefore, anxiolytic effects of *A.* *millefolium* hydroalcoholic extract were not mediated by GABA (A)/ benzodiazepine (BDZ) neurotransmission and did not lead to tolerance following short-term, repeated administration (Baretta et al., 2012).

*A. millefolium* (8.0, 10.0 or 12.0 mg/kg) decreased conflict behavior during late proestrus but, during diestrus, 12.0 mg/kg of the plant reduced conflict behavior (Molina‐Hernandez et al., 2004). Polar-fraction of *A. millefolium* showed higher sedative, pre-anesthetic and anti-anxiety effects than diazepam. 

Hydroalcoholic extract of *A. wilhelmsii* (100, 200, and 400 mg/kg) increased NO metabolites concentrations in the hippocampal tissues and affected the severity of seizures in pentylenetetrazole-induced seizure model (Hosseini et al., 2014). Table 4 summarizes the effects of *Achillea* species plants on the cardiovascular, endocrine and nervous systems.

### Clinical effects

In a trial (triple-blind randomized placebo-controlled), *A. millefolium* (250 and 500 mg/day, for one year), reduced annual relapse rate in multiple sclerosis (MS) patients and the mean volume of lesions was diminished by 500 mg of *A. millefolium*. It decreased the expanded disability status scale score and improved performance in word-pair learning, paced auditory serial addition task, and Wisconsin card sorting test, (Ayoobi et al., 2019). *A. millefolium* distillate, 4 times a day for 14 days, in a double-blind randomized controlled trial, healed chemotherapy-induced oral mucositis in cancer patients (Miranzadeh et al., 2015).

In a clinical trial, treatment with hydroalcoholic capsules of *A. millefolium* (150 mg/8 hr) in the first three days of menstruation for two menstrual cycles, reduced menstrual pain severity (Radfar et al., 2018). Treatment with *A. millefolium* form the 3rd day in 2 menstruation cycles minimized the pain severity in primary dysmenorrhea (Jenabi and Fereidoony, 2015). Treatment with herbal combination of *Eleutherococcus senticosus*, *Achillea millefolium*, and *Lamium album* for 2 weeks in patients with atopic dermatitis had no advantage over placebo (Shapira et al., 2005).

### Possible mechanisms of the effects of Achillea plants

#### Anti-inflammatory effects

Polyunsaturated alkamides constituents of *Achillea* species inhibit activity on cyclooxygenase and 5-lipoxygenase *in vitro*, which appeared to be dependent on the particular structure of the alkamides (Abdalla et al., 1989). *A. millefolium* is mainly known for its anti-inflammatory effects (Akram, 2013). The plant is traditionally used for treatment of hepato-biliary disorders, gastro-intestinal and as an antiphlogistic drug (Benedek et al., 2007). 

Benedek et al. investigated the impact of *A. millefolium* plant extract on protease inhibition *in vitro* to comprehend its anti-inflammatory action. The extract and flavonoid fraction inhibited human neutrophil elastase, while the dicaffeoylquinic acid (DCQA) fraction showed less activity. Matrix metalloproteinases (MMPs) were also inhibited, with the DCQA fraction which had stronger effects. Hence, *A. millefolium*'s *in vitro* antiphlogistic activity may be partially mediated by inhibition of human neutrophil elastase (HNE) and MMP-2 and -9 (Benedek et al., 2007).

An aqueous extract of *A. millefolium* dry flower showed anti-inflammatory activity, as shown by the mouse paw edema test. Fractionation isolated a water-soluble material that reduced inflammation by 35%. Studies have shown that this fraction is made up of protein-carbohydrate complexes (Goldberg et al., 1969).

Ethanol extract of* A. millefolium* (50%) reduced the expression of pro-inflammatory cytokines, such as iNOS, COX-2, and IL-6 in lipopolysaccharide (LPS)-treated murine macrophage Raw 264.7 cells, indicated anti-atopic dermatitis activity of the plant (Ngo et al., 2020).

*A. santolina* has been traditionally used as an anti-inflammatory remedy and to relieve pain (Al-Snafi, 2013). Tekieh et al. demonstrated that *A. santolina* extract reduced edema, hyperalgesia, and serum IL-6 levels in complete Freund's adjuvant (CFA)-induced inflammation in rats (Tekieh et al., 2011). Zaringhalam et al found that* A. santolina* extract had anti-hyperalgesic and anti-inflammatory effects with pretreatment and short-term treatment (Zaringhalam et al., 2010).

The extract of *A. ageratum* and its components showed greater effectiveness in the acute phase of tetradecanoylphorbol acetate (TPA)-induced mouse ear edema compared to chronic phase indicated by neutrophil migration inhibition and myeloperoxydase activity (Gomez et al., 1999).

The derivatives isolated from methylene chloride-methanol extract of aerial parts of *A. coarctata* enhanced the proliferation of macrophages and exhibited anti-inflammatory properties (Hegazy et al., 2008).

In LPS-stimulated NR8383 macrophages, zaluzanin D from *A. acuminate* reduced nitric oxide (NO) production and inflammatory cytokine secretion. Zaluzanin D also reduced macrophage infiltrations and inflammatory changes in lung tissues in LPS-induced rats. Additionally, zaluzanin D inhibited lipid peroxidation, recruited anti-oxidative defense system, and regulated TNF-α, IL-1β, and IL-6 levels in the lungs by inhibiting NF-kB pathway (Tong et al., 2021).

#### Anti-oxidant effects

*A. falcata* was the most effective species as antioxidant enzyme activities in erythrocytes, *A. crithmifolia* and *A. nobilis* subsp. neilrechii showed the highest activities on CAT in leucocytes, while *A. millefolium* subsp. pannonica, *A. teretifolia*, and *A. nobilis* subsp. sipylea had more marked effects on SOD, glutathione peroxidase (GPx), and lactoperoxidase (LPO) enzyme (Konyalioglu and Karamenderes, 2005).

In the livers of diabetic rats,* A. santolina* extracts improved protein oxidation, lipid peroxidation, and antioxidant defense system and reduced liver malondialdehyde (MDA) and protein carbonyls but increased glutathione (GSH), SOD, and CAT levels. The extract also reduced serum glucose levels and modulated, aspartate transaminase (AST), alanine transaminase (ALT), and alkaline phosphatase (ALP) in diabetic rats, suggesting a possible correlation between hypoglycemic and antioxidant activity (Ardestani and Yazdanparast, 2006).

*A. millefolium* extract due to the presence of phenolic compounds, showed antiradical activity and decreased H_2_O_2_ production in isolated mitochondria and State 3 respiration rate in rat heart mitochondria (Trumbeckaite et al., 2011).

Methanolic extract of *A. biebersteinii* showed highest but inflorescence extract of *A. eriophora* showed lowest DPPH radical scavenging activity. *A. biebersteinii *leaves extract pretreatment was more effective than *A. eriophorea* in inhibition of Human Foreskin Fibroblast (HFF3) injuries caused by H_2_O_2_ treatment (Varasteh-Kojourian et al., 2017).

Glutathione S-transferase (GST), α-glycosidase (α-Gly), and butyrylcholinesterase (BChE) enzymes were also inhibited by *A. schischkinii* methanolic extract (Türkan et al., 2020).

#### Immunosuppressive effects

Administration of *A. talagonica* extract to mice, prior to immunization with sheep red blood cells (SRBC), resulted in a significant dose-dependent decrease in hemagglutination antibody (HA) titer. After intra-scapular injection of 0.5 g/kg, in primary response, rabbits showed a significant decrease in titer of total antibody to hepatitis D antigen (anti-HD), but no changes were observed in secondary response. This suggests that the immunosuppressive activity of *A. talagonica*, particularly affects humoral immunity (Rezaeipoor et al., 1999).

The immunosuppressive property of choline, a constituent of *A. talagonica* was similar to that of prednisolone (5 and 10 mg/kg). Additionally, quercetin and caffeoyl glucoside (both, 20 mg/kg) decreased the anti-SRBC titer compared to the control group (Saeidnia et al., 2015). The anti-SRBC titer in mice was decreased by the volatile oil of *A. millefolium*. The different immunological effects of *A. millefolium* and *A. talagonica* could be due to their constituents, sesquiterpenes and proazulene (Saeidnia et al., 2004).

Mainly glycosylated derivatives of caffeic acid from *A. millefolium* decreased the anti-SRBC titer in mice (Yassa et al., 2007). *A. wilhelmsii* aqueous extract (100 mg/kg) significantly increased the delayed type of hypersensitivity response in mice and in the haemagglutination titer test, the extract (50 mg/kg) showed a stimulatory effect. Therefore, a stimulatory effect of *A. wilhelmsii* on both humoral and cellular immune functions was shown (Sharififar et al., 2009).

The immune-protective effects of *A. fragrantissima* oil extract was demonstrated in mice by improvement in the haemagglutination index, reduced feet swelling, and increased spleen weight (Al-Sarraf et al., 2020). Anti-inflammatory, anti-oxidant, immunosuppressive, antitumor, antimicrobial and wound-healing properties of *Achillea* species plants are summarized in Table 2.

## Discussion

The current article reviewed various pharmacological effects and possible molecular mechanism of *Achillea* species in both experimental and clinical investigations. Studies from 1969 to 2021 revealed a wide range of pharmacological effects for these plants. Immunosuppressive, anti-inflammatory and anti-oxidant effects were shown for these plants. In addition, it was shown that these plants pose wound-healing and antimicrobial effects on various Gram positive and Gram-negative bacteria as well as antitumor activity on different cell lines. The antispasmodic effects of the plants and their constituents were also demonstrated on different smooth muscle types. The effect of the plants on gastrointestinal including hepatoprotective and gastroprotective was also reported. *Achillea* species also showed anti-arrhythmic, anti-thrombotic, vaso-relaxant, anti-hyperlipidemic and anti-hypertensive effects. In addition, the plants showed different endocrine effects such as anti-diabetic, estrogenic and anti-spermatogenic properties. Neurological effects of the plants include anti-nociceptive and anti-anxiety activity. Table 5 describes the possible molecular mechanisms of some of pharmacological actions of the genus *Achillea*.

**Table 1:** Chemical constituents of the plants in the genus *Achillea*

	Compound classes	Compound Name		
Terpenoids	Monoterpenes	Santolinanes	8-Hydroxysantolina-1,4-dien-6-al 1-Santolinene-4,5,8-triol 5,8-Epoxy-4,6-dihydroxysantolin-1-ene	
	Sesquiterpenes	12,6a-Guaianolides	Achillicin Leucodin Matricarin Rupicolin A and B Chrysartemin A Isoapressin Apressin	
		Nor-Guaianolides	3-Oxaachillicin Crithmifolide Achilleppolide Chamazulene	
		12,8a-Guaianolides		
		1(10!9)-Abeo-12,8a-Guaianolides	Acrifolide Ligustolide A and B Tauremisin Arglanin Artecalin Santamarin Reynosin	
		Eudesmanes	12,6a-eudesmanolides eudesmanes	
		Germacranes	Ageratriol Ridentin Sintenin Artabin Achillolide A and B	
		Bisabolanes		
		Elemanes	b-Elemen-9b-ol	
		Oplopanes	7b-Hydroxy-11-oplopenone	
			3a,7a,11-Trihydroxycyperan-4-one	
		Longipinanes	a-Longipin-2-en-1-one	
			7b-Hydroxy-a-longipin-2-en-1-one	
			5-Hydroxy-5,6-seco-caryophyllen-6-one	
		Farnesanes	9-Hydroxyfarnesyl acetate	
			w-Oxonerolidol	
	Diterpenes	16a,17-Epoxy-ent-kaurane		
		16a,17-Epoxy-19-acetoxy-ent-kaurane		
		16a,17-Epoxy-3a-acetoxy-ent-kaurane		
	Triterpenes	Achilleol A		
		Achilleol B		

Lignans	3’-Demethoxyaschantin			
	Epiaschantin			
	Aschantin			
	Episesartemin			
	Sesartemin			
	Epieudesmin			
	Epiyangambin			
	Yangambin			
	3’-Demethoxyyangambin			
	Iso-3’-Demethoxyyangambin			
Flavonoids	Luteolin	Isoschaftoside		Cirsimaritin
	Cynaroside	Vicenin-2		Pectolinarigenin
	Luteolin 7-malonylglucoside	Vitexin		Salvigenin
	Apigenin	Orientin		Nepetin
	Cosmosiin	Isoorientin		Axillarin
	Apigenin 7-malonylglucoside	Isoorientin 7-methyl ether		Jaceidin
	Rutin	Penduletin		Centaureidin
	Chrysoeriol	Chrysosplenol D		Chrysosplenetin
	Desmathoxycentauridin	Luteolin 4’-glucoside		Casticin
	Quercetin	Hispidulin		Eupatolin
	Quercetin 3-methyl ether	Cirsiliol		6-Demethoxycapillarisin
	Quercetin 3,3’- dimethyl ether	Santoflavone		3-Methylbetuletol
	5-Hydroxy-3,6,7,4’ tetramethoxyflavone	5-Hydroxy-3,6,7,3’,4’- Pentamethoxyflavone		6-Hydroxykaempferol 3,6-dimethyl ether
	Schaftoside	Isoorientin 7,3’- dimethyl ether		6-Hydroxykaempferol 3,6,7,4’-tetramethyl ether
Amino acid derivatives	Choline			
	Betaine			
	Proline			
	Stachydrin			
	Betonicine			
Fatty acids				
Alkanes				
Inulin				

**Table 2:** Anti-inflammatory, anti-oxidant, immunosuppressive, antitumor, antimicrobial and wound-healing effects of *Achillea* species plants

Effects	*Achillea* species	Extract	Constituents/ fractions	Ref.
Wound healing	*A. kellalensis*	AE of flowers		(Pirbalouti et al., 2010)
	*A. biebersteinii*	HE of aerial parts	Sesquiterpenoid β-Eudesmol, piperitone, camphor, borneol, α-terpinene, 1, 8-cineole.	(Akkol et al., 2011)
	*A. eriophora*	ME		(Varasteh-Kojourian et al., 2017)
	*A. asiatica*	EE	Luteolin; and apigenin	(Dorjsembe et al., 2017)
	*A. millefolium*	AEE		(Hemmati et al., 2002; Temamogullari et al., 2009)
		EE and AE		(Nirmala and Karthiyayini, 2011)
Immunosuppressive	*A. talagonica*	ME and AME	Caffeic acid 9-O-glucoside, quercetin, luteolin, 3'-methoxy luteolin, proline, and choline.	(Saeidnia et al., 2015)
	*A. millefolium*	EO	bisabolol XXVI	(Saeidnia et al., 2004)
		ME	Caffeic acid glucoside XXII	(Yassa et al., 2007)
	*A. wilhelmsii*	AE		(Sharififar et al., 2009)
	*A. fragrantissima*	EO		(Al-Sarraf et al., 2020)
Anti-inflammatory	*A. millefolium*	CE	Flavonoids and dicaffeoylquinic acids fractions	(Benedek et al., 2007)
		AE of the dry flower	Nonsteroidal	(Goldberg et al., 1969)
		EE		(Ngo et al., 2020)
	*A. santolina*	ME		(Tekieh et al., 2011)
	*A.* *ageratum*	ChE	Stigmasterol and ß-sitosterol	(Gomez et al., 1999)
	*A. coarctata*	MCME of aerial parts	1α,6α,8α-trihydroxy-5α,7βH-guaia-3,10,11-trien-12-oic acid XXXVI; 1α,6α,8α-trihydroxy-5α,7βH-guaia-3,9,11-trien-12-oic acid XXXVII; ligustolide-A XXXVIII; arteludovicinolide-A XXXIX and austricin XL	(Hegazy et al., 2008)
	*A. acuminate*		Zaluzanin D	(Tong et al., 2021)
Anti-oxidant	*A. santolina*	AEE		(Ardestani and Yazdanparast, 2006)
	*A.* *millefolium*	AEE of air-dried aerial parts		(Trumbeckaite et al., 2011)
	*A. crithmifolia* *A. nobilis* *A. millefolium* *A. teretifolia* *A. nobilis* *A. falcate* *A. setacea*	Dried and pulverized flower heads of plants were boiled in distilled water		(Konyalioglu and Karamenderes, 2005)
	*A. biebersteinii* *A. eriophora*	ME of leaf and inflorescence	Phenol and flavonoid	(Varasteh-Kojourian et al., 2017)
	*A. schischkinii*	AE, ME		(Türkan et al., 2020)
Antimicrobial	*A. damascena*	ME of whole plant		(Barbour et al., 2004)
	*A. clavennae* *A. holosericea* *A. lingulata* *A. millefolium*	The extracts of the aerial parts	Alkanes, fatty acids, monoterpenes, the guaiane sesquiterpenes and flavonoids.	(Stojanović et al., 2005)
	*A. millefolium*	EO		(Boris et al., 2021; Candan et al., 2003; Daniel et al., 2020)
			Luteolin, apigenin, centaureidin, and nevadensin	(Salomon et al., 2021)
	*A. santolina*	CE		(Khalil et al., 2009)
	*A. biebersteinii*			
		EO		(Bariş et al., 2006)
			biebersteiniside XXIX; 6-epiroseoside XXX; ascaridole XXXI; strictic acid XXXII; and centipedic acid XXXIII	(Al-Shuneigat et al., 2020; Mahmoud et al., 2006)
	*A. falcate*	EE and EO		(Khalil et al., 2009)
		EO and ME	1, 8-cineole; camphor; and borneol	(Candan et al., 2003)
	*A. setacea*	EO from air-dried aerial parts	1, 8-Cineole	(Unlu et al., 2002)
	*A. teretifolia*			
	*A. wilhelmsii*	EO	Thymol (65%) and carvacrol (19%)	(Kazemi and Rostami, 2015)
	*A. gypsicola*	EO	camphor, 1,8-cineol and borneol	(Açıkgöz, 2020)
	*A. filipendulina*	EO	Neryl acetate, spathulenol, carvacrol, santolina alcohol, trans‐caryophyllene oxide, 1,8‐cineole, camphor, ascaridole, trans‐isoascaridole, piperitone oxide, ascaridole, and p‐cymene	(Aminkhani et al., 2020)
Antitumor	*A. clavennae*		Centaureidin	(Si et al., 2006)
			Guaianolides; 9α-acetoxyartecanin XVII; apressin XVIII; inducumenone XIX; and centaureidin XX	(Trifunović et al., 2006)
	*A. falcata*		3β-methoxy-iso-seco-tanapartholide XIII; tanaphillin XIV; iso-seco-tanapartholide XV; and 8-hydroxy-3-methoxy-iso-seco-tanaparatholide XVI	(Ghantous et al., 2009)
	*A.* * millefolium*	ChE of the aerial parts	Centaureidin	(Csupor‐Löffler et al., 2009b)
		AE, EE and ME		(Amini Navaie et al., 2015)
			ethyl acetate fraction	(Abou Baker, 2020)
	*A. wilhelmsii*	AEE		(Sargazi et al., 2020)
	*A. ageratum*	ChE	Stigmasterol and β-sitosterol	(Gómez et al., 2001)
	*A. fragrantissima*	EO		(Choucry, 2017)
	*A. membranacea*	EO	1,8-cineole	(Abdalla et al., 2020)

Abbreviations: AE: aqueous extract, HE: hexane extract, ME: methanol extract, EE: ethanol extract, AEE: aqueous-ethanol extract, AME: aqueous-methanol extract, EO: essential oil, CE: crude extract, ChE: chloroform extract, MCME: methylene chloride-methanol extract.

**Table 3:** Smooth muscle relaxant effects of *Achillea* species plants and their effects on the digestive system

Effects	*Achillea* species	Extract	Constituents/ fractions	Ref.
Antispasmodic	*A. nobilis* subsp. Sipylea	Total extract		(Karamenderes and Apaydin, 2003)
	*A. millefolium*	EE and AEE of flower	5-aminoindazole, gossypol and *Trypterygium wilfordii*	(Montanari et al., 1998)
		AEE	Quercetin and apigenin	(Sedighi et al., 2013)
	*A.* *nobilis*		5-hydroxy-3,6,7,4′-tetramethoxyflavone	(Kastner et al., 1995)
	*A. fragrantissima*		Cirsiliol	(Mustafa et al., 1992)
Relaxant	*A. wilhelmsii*		Carvacrol	(Boskabady et al., 2009)
	*A. millefolium*	AEE		(Koushyar et al., 2013)
		HE		(Arias-Durán et al., 2020)
Hepatoprotective	*A.* *millefolium*	CE		(Yaeesh et al., 2006)
Anti-ulcerogenic	*A. millefolium*	ME		(Mahady et al., 2005)
		AE		(Baggio et al., 2002)
	*A.wilhelmsii*	AEE		(Niazmand et al., 2010)
Gastroprotective	*A. millefolium*	AEE		(Potrich et al., 2010)
Inhibition of gastric acid output	*A. wilhelmsii*	AEE		(Niazmand et al., 2010)
Choleretic	*A.* *millefolium*	SE	Dicaffeoylquinic acids (DCCAs) and luteolin fractions	(Benedek et al., 2006)
Orexigenic	*A.* *millefolium*	AEE		(Nematy et al., 2017)

Abbreviations: AE: aqueous extract, HE: hexane extract, ME: methanol extract, EE: ethanol extract, AEE: aqueous-ethanol extract, AME: aqueous-methanol extract, EO: essential oil, CE: crude extract, ChE: chloroform extract, MCME: methylene chloride-methanol extract, SE: solidphase extract.

**Table 4:** The effects of *Achillea* species plants on the cardiovascular, endocrine and nervous systems

Effects	*Achillea* species	Extract	Constituents/ fractions	Ref.
Anti-arrhythmic effect	*A. santolina*	ME		
Anti-aggregant and antithrombotic effect		CE of Leaf	Fractions extracted with chloroform (F1), diethyl ether (F2), ethyl acetate (F3) and water (F4)	(Al-Awwadi, 2010)
Vasoprotective activity	*A.* *millefolium*	ME of aerial parts of plant	Flavonoids (10%) and dicaffeolylquinic acid derivatives (12%)	(Dall’Acqua et al., 2011)
		EE		(Eslamifar and Sabbagh, 2020)
Anti-hyperlipidemic effect	*A. wilhelmsii*	AEE of air-dried powder from aerial parts of flowers		(Asgary et al., 2000)
Anti-hypertensive effect	*A. wilhelmsii*	AEE	Carvacrol, luteolin, apigenin and 1,8-cineole	(Niazmand et al., 2011)
Antispasmodic and vasorelaxant effects	*A. wilhelmsii*		Thymol and carvacrol	(Peixoto‐Neves et al., 2010)
			Luteolin	(Jiang et al., 2005; Qian et al., 2010)
			Carvacrol	(Boskabady and Jandaghi, 2003; Can Baser, 2008; Peixoto‐Neves et al., 2010)
			Apigenin	(Jin et al., 2009)
			1, 8-cineole	(Lahlou et al., 2002; Nascimento et al., 2009)
		AEE		(Niazmand et al., 2014)
Anti-diabetic activity Hypoglycemic effects	*A. santolina*	AE		(Al-Snafi, 2013; Yazdanparast et al., 2007)
Estrogenic activity	*A.* *millefolium*		Dihydrodehydrodiconiferyl alcohol 9-O-beta-D-glucopyranoside, apigenin and luteolin	(Innocenti et al., 2007)
Anti-spermatogenic effect	*A.* *millefolium*	AEE		(Montanari et al., 1998)
		Inflorescence AEE		(Karimpour et al., 2020)
	*A. santolina*	AEE		(Golalipour et al., 2004)
Anti-nociceptive activity	*A.* *millefolium*	AEE		(Pires et al., 2009)
Anti-anxiety effect			Polar fraction	(Rezaie and Ahmadizadeh, 2013)
		AEE		(Baretta et al., 2012)
Anticonflict-like actions		AEE		(Molina‐Hernandez et al., 2004)
Sleeping aids			Fraction, containing α-acids	(Zanoli et al., 2005)
Antiseizure	*A. wilhelmsii*	AEE		(Hosseini et al., 2014)

Abbreviations: AE: aqueous extract, HE: hexane extract, ME: methanol extract, EE: ethanol extract, AEE: aqueous-ethanol extract, AME: aqueous-methanol extract, EO: essential oil, CE: crude extract, ChE: chloroform extract, MCME: methylene chloride-methanol extract.

**Table 5:** The possible molecular mechanisms of some of pharmacological actions of the genus *Achillea*.

Pharmacological action	Genus *Achillea*	Possible molecular mechanisms
Anti-inflammatory	*A. millefolium*	HNE inhibition MMP-2 and -9 inhibition
	*A. santolina*	↓ IL-6
	*A. coarctata*	Proliferation of beneficial macrophages
Anti-oxidant	*A. crithmifolia* *A. nobilis*	↑ CAT
	*A. millefolium*	↑ SOD
	*A. teretifolia*	↑ GPx
	*A. nobilis*	↑ LPO
	*A. santolina*	↓ serum glucose ↓ MDA ↓ PCO Modulation of ALP, ALT and AST
	*A. millefolium*	↓ H2O2
Immunosuppressive	*A. talagonica*	↓ HA titer ↓ anti-HD titer
Antitumor Anti-proliferative	*A. falcata*	↓ keratinocyte cell viability
	*A. fragrantissima*	Interference with cell growth
Wound healing	*A. asiatica*	↓ NO ↓ PGE2 ↓ TNF-α ↓ IL-1β ↓ IL-6 ↓ COX-2 Activation of TGF-β Stimulation of collagen expression Induction of β-catenin and Akt Stimulation of keratinocyte differentiation and migration
Smooth muscle Relaxant	*A. nobilis* *A. wilhelmsii*	Inhibition of transmembrane Ca^2+^ influx
	*A. millefolium *	Inhibition of muscarinic receptor Stimulation of β2-adrenergic receptors Inhibition of histamine (H1) receptors
Anti-ulcerogenic	*A. millefolium*	Inhibition of gastric secretion Increase in protective factors (blood flow)
Anti-hypertensive	*A. wilhelmsii*	Inhibition of sarcolemmal Ca^2+^ channels Inhibition of intracellular calcium release Activation of K^+ ^channels Inhibition of extracellular Ca^2+^ influx via VDDCs and ROCCs
lowering blood lipid properties	*A. wilhelmsii*	↓ TG ↓ Chol ↓ LDL ↑ HDL
Anti-diabetic	*A. santolina*	↓ serum glucose ↓ NO ↓ MDA ↓ PCO ↓ AOPP ↑ GSH ↑ CAT ↑ SOD
Estrogenic	*A. millefolium*	Stimulation of α and β receptors of estrogen
Anti-spermatogenic	*A. millefolium*	Increased number of metaphases in the germ epithelium
	*A. santolina*	Disorganized germ epithelium Exfoliation of immature germ cells Germ cell necrosis Increased number of metaphases in germinal epithelium of seminiferous tubules
Anti-nociceptive	*A. millefolium*	Central adenosine mechanism Inhibition of GAD activity Interact with either GAD or GABA-T Ultimately influence brain GABA levels and neurotransmission

Abbreviations: HNE: human neutrophil elastase, MMP: matrix metalloproteinases, IL-6: interleukin 6, CAT: catalase, SOD: superoxide dismutase, GPx: glutathione peroxidase, LPO: lactoperoxidase, MDA: malondialdehyde, PCO: protein carbonyls, ALP: alkaline phosphatase, ALT: alanine transaminase, AST: aspartate transaminase, HA: hemagglutination antibody, NO: nitric oxide, PGE2: prostaglandin E2, TNF-α: tumor necrosis factor-alpha, IL-1β: interleukin 1 beta, COX-2: cyclooxygenase-2, VDDCs and ROCCs: voltage and receptor operated calcium channels, TG: triglyceride, Chol: cholesterol, LDL: low-density lipoprotein, HDL: high-density lipoprotein, AOPP: advanced oxidation protein products, GSH: glutathione, GAD: glutamic acid decarboxylase, GABA-T: gamma-Aminobutyric acid transaminase.

Clinical studies also indicated therapeutic effect of *A. millefolium *on MS, chemotherapy-induced oral mucositis in cancer patients, and dysmenorrhea but not on atopic dermatitis. 

Therefore, *Achillea* species could be of therapeutic potential for treating of a wide range of diseases. However, there are still several aspects of *Achillea* plants that have received little attention so far. Therefore, further studies are needed to evaluate its phytochemical, biological and especially clinical effects of this genus. In addition, molecular mechanisms of the effects of these plants and their constituents should be studied in the future.
